# An Uncertainty Modeling Framework for Intracardiac Electrogram Analysis

**DOI:** 10.3390/bioengineering7020062

**Published:** 2020-06-26

**Authors:** Amirhossein Koneshloo, Dongping Du, Yuncheng Du

**Affiliations:** 1 Department of Industrial, Manufacturing and Systems Engineering, Texas Tech University, Lubbock, TX 79409, USA; amir.koneshloo@ttu.edu; 2 Department of Chemical & Biomolecular Engineering, Clarkson University, Potsdam, NY 13699, USA; ydu@clarkson.edu

**Keywords:** statistical modeling, maximum likelihood estimation, intracardiac electrogram analysis, uncertainty analysis

## Abstract

Intracardiac electrograms (EGMs) are electrical signals measured within the chambers of the heart, which can be used to locate abnormal cardiac tissue and guide catheter ablations to treat cardiac arrhythmias. EGMs may contain large amounts of uncertainty and irregular variations, which pose significant challenges in data analysis. This study aims to introduce a statistical approach to account for the data uncertainty while analyzing EGMs for abnormal electrical impulse identification. The activation order of catheter sensors was modeled with a multinomial distribution, and maximum likelihood estimations were done to track the electrical wave conduction path in the presence of uncertainty. Robust optimization was performed to locate the electrical impulses based on the local conduction velocity and the geodesic distances between catheter sensors. The proposed algorithm can identify the focal sources when the electrical conduction is initiated by irregular electrical impulses and involves wave collisions, breakups, and spiral waves. The statistical modeling framework can efficiently deal with data uncertainties and provide a reliable estimation of the focal source locations. This shows the great potential of a statistical approach for the quantitative analysis of the stochastic activity of electrical waves in cardiac disorders and suggests future investigations integrating statistical methods with a deterministic geometry-based method to achieve advanced diagnostic performance.

## 1. Introduction

Cardiac disease is the leading cause of death in U.S. and in the world [1]. Cardiac arrhythmia is a common cardiac disorder caused by irregular heartbeats initiated in different chambers of the heart. Supraventricular Arrhythmias such as Atrial Fibrillation (AF) and Atrial Flutter are very common irregular heart rhythms [2]. This group of conditions occurs when abnormal electrical impulses take the control of the heart rhythm from the normal sinus node pacemaker and initiate rapid and irregular activities in different areas of atria, which cause the atria to quiver [3]. Treatments of supraventricular arrhythmias include the antiarrhythmic medication to control heart rate and rhythm and prevent blood clotting, as well as radiofrequency catheter ablation (RFA) that ablates abnormal electrical pathways in atrial tissue. RFA is more effective than antiarrhythmic drug therapy for patients with recurrent symptoms and is frequently used when the medication therapy initially fails [4,5]. RFA is guided by the intracardiac electrograms (EGMs), where the recordings are analyzed to identify the drivers (rotor, foci, and breakthrough) that cause and maintain abnormal electrical activities.

There have been many advances in EGM analysis to characterize the electrical activities in abnormal cardiac rhythms. Richter et al. analyzed the propagation patterns of electrical waves during AF using EGM through sparse modeling [6]. Cantwell et al. [7] surveyed the algorithms designed for identifying local activation times (LATs) and calculating the conduction velocity (CV) of electrical waves, and pointed out that estimating the uncertainty associated with the CV computation remains a challenge. In addition, the dominant frequency (DF) mapping method was used in a few studies to localize abnormal pacing sites of high frequency during AF [8,9,10]. However, Salinet et al. showed that targeting the ablation site using DF could be unreliable [11]. Later, Li et al. developed an integrated 3D software platform combining the mapping of both frequency spectral and phase from EGMs to guide persistent AF [12]. Furthermore, causality analysis and DF are combined to estimate the propagation pattern of electrical waves from EGMs, which is then used to identify the area responsible for arrythmia [13]. Guillem et al. showed that the DF method provides more reliable results in comparison with the phase singularity method. However, in the absence of Left-Atrium-to-Right-Atrium gradient, the accuracy of the DF-based methods is not guaranteed [14]. 

In addition, signal processing and data mining are also examined as useful approaches to map the electrical activities in arrythmia from EGMs. For example, classification methods such as Linear Discriminant Analysis (LDA) and Quadratic Discriminant Analysis (QDA) were used to predict the origins of abnormal electrical impulses [15,16]. Additionally, the stochastic trajectory analysis of ranked signals (STAR) mapping has been used to map arrhythmia from EGM data [17], and a recent study combined DF analysis with STAR mapping and suggested that the early sites of activation correlate with rapidity and organization markers [18]. Some studies utilized data mining approaches to detect the unsteady dynamics of EGMs. Specifically, independent component analysis (ICA) is integrated with the second order blind identification (SOBI) to find the main patterns of atrial activities in AF [19]. Orozco-Duque et al. computed the morphological and non-linear features from EGMs and used these features in a semi-supervised clustering approach to locate the critical AF sites. Cervigón et al. used principle component analysis and the Granger causality and divergence technique to analyze the connectivity between two atrial chambers and pulmonary veins [20]. In addition, recurrence quantification analysis (RQA) is conducted to obtain a better picture of the disorganized patterns of intracardial signals [21]. Almeida et al. characterized the dynamics of atrial tissue activations from fractionated EGMs collected during persistent AF using recurrence plots and recurrence quantification analysis (RQA), and showed that the RQA variables were effective in discriminating normal vs. fractionated EGMs [22]. 

Apart from focusing exclusively on the information provided by the electrograms, there are different perspectives applied to extract information from EGMs. In the study by Weber et al., iterative optimization is done to predict the earliest activation using LATs within a 3-dimensional anatomic map of heart chamber [23]. Many studies have been done to locate the AF focal source [24,25,26], where the geodesic information is combined with EGMs to infer the electrical activities in arrythmia. Ganesan et al. designed an algorithm to map the region of rotor and tested their algorithm on simulated two-dimensional heart tissue. Moreover, a recently published work by Gaeta et al. discussed the directional sensitivity of bipolar electrode and suggested that the rotation of the electrode pair can affect the amplitude of EGMs [27]. Moreover, focal impulse and rotor modulation (FIRM) is another effective method that provides a mechanistic framework for AF mapping and ablation [28,29]. Alhusseini et. al. implemented the FIRM method to identify the rotational activation patterns from EGMs and targeted the ablation at these rotational sites which terminated AF to sinus rhythm for 10 out of 12 patients [30]. 

The previous works have contributed a great amount of knowledge in EGM analysis. However, data uncertainty and their impacts on the analysis have not been discussed much. During arrythmia, normal electrical waves may collide with the abnormal waves triggered by different drivers and further evolve into spiral waves or break into small wavelets. In this case, activations measured in the EGMs are often in messy orders. As a result, diagnosis based on the activation orders can be misguided. In addition, due to the chaotic activities of the electrical waves and the presence of measurement noise, LAT estimations can be inaccurate. Moreover, the activation order recorded in EGMs may be affected by the spatial location of the sensor with respect to the focal source location. Therefore, when the sensor position changes, the estimated conduction pattern will be affected. This will introduce another source of uncertainty to the signal processing and data analysis. Finally, there is an uncertainty associated with the estimation of CV due to the variations in cardiac muscle fibers and measurement noise. For the geometry-based method, CV is important information to infer the electrical activities inside the heart chambers. Without considering the uncertainty in CV, detection algorithm may draw incorrect conclusions.

To address the abovementioned uncertainties, this paper designed a new probabilistic approach, which integrates statistical modeling with robust optimization to determine the propagating pattern of electrical waves and further suggest a region that contains ectopic pacemakers considering the uncertainty in CV estimation. The contribution of this study is to introduce the concept of statistical modeling and uncertainty analysis in EGM analysis and provide more reliable estimation of LAT and CV for mapping the electrical activities in cardiac chambers. The rest of the paper is organized as follows. Section 2 describes the method proposed in this study, and Section 3 presents the design of the experiment for simulation study and algorithm validation. Section 4 includes experimental results and discussions.

## 2. Methods

In this section, a multinomial distribution model will be first defined to describe the activation orders of catheter electrodes, then a robust maximum likelihood estimation is formulated to find the probability that each pair of electrodes activates first or last. To ensure a better estimation of activation order, a robust probability boundary is computed, which is followed by decision-making to determine focal source locations. 

**Figure 1:**
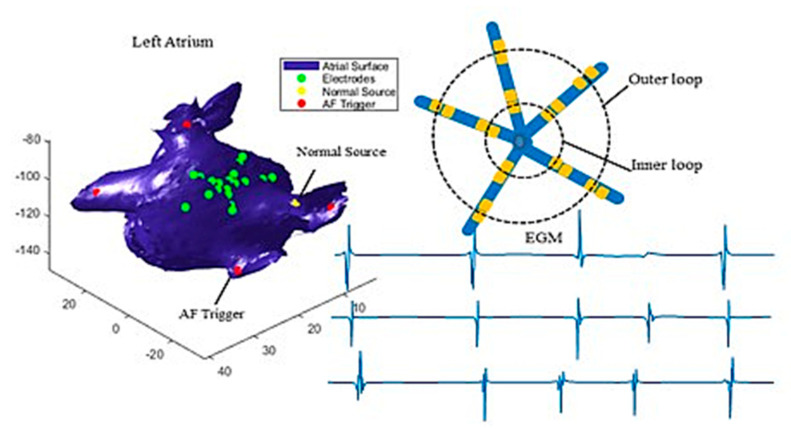
Illustration of simulated catheter and intracardial electrogram in human left atrium.

In this paper, a PentaRay catheter was simulated to illustrate the proposed method. However, the algorithm can be generalized to different types of catheters. Figure 1 illustrates how the catheter is defined. The device contains 20 electrodes which are spaced along five branches. The 20 electrodes are grouped into two loops, i.e., the outer loop (bigger circle) and the inner loop (smaller circle), where each loop contains five pairs of electrodes. Each pair of electrodes measures one channel of EGM signals. Figure 1 shows three simulated EGMs measured at three pairs of electrodes. Here, we only present three recordings as examples, but our analysis used all ten recordings.

To account for the uncertainty in LATs, it is assumed that the true activation is observed with errors as $x_{i,j}\left(t\right)\;∿\;N\left(x_{i,j}^{EGM}\left(t\right),\sigma^{2}\right)$, where $x_{i,j}\left(t\right)$ is the true activation time of the electrode pair j, j = 1,…, D located in loop i, i = 1,2 after an electrical wave t enters the loop from an unknown source, where t represents the tth independent activation. $x_{i,j}^{EGM}\left(t\right)$ is the time corresponding to maximum change in the signal at activation t. The true LAT follows a normal distribution with a mean of $x_{i,j}^{EGM}\left(t\right)$ and variance $\sigma^{2}$. Suppose that a set of times $X_{i,j}$ = $\left\{x_{i,j}\left(1\right),\;\ldots ,x_{i,j}\left(T\right)\right\}$ is sampled from the distribution of LAT, which denotes the activation times of T independent activations.

### 2.1. Multinomial Distribution

When an electrical wave passes through one of the loops, regarding which pair of sensors in a loop activates first or last, there are only D possible outcomes. It means only one of the D pairs of sensors can be activated first or last. Alternatively, it can be stated that the event of a pair of electrodes gets activated first or last follows a Bernoulli trial, such that the probability of success can be interpreted as the probability of the pair being activated first or last once the wave enters the loop. Hence, one can show that this view of the system leads to the definition of multinomial distribution, where each activation has D possible outcomes. Therefore, the probability distribution function of the multinomial distribution given T independent activations for D pairs of electrodes in loop i is given as follows:

(1) $$\begin{array}{c} f\left(n_{i,1},\ldots ,n_{i,D}|T,p_{i,1},\ldots ,p_{i,D}\right)=Pr\left(N_{i,1}=n_{i,1},\ldots ,\;N_{i,D}=n_{i,D}\right)\\ =\frac{n!}{n_{i,1}!n_{i,2}!\ldots n_{i,D}!}p_{i,1}^{n_{i,1}}p_{i,2}^{n_{i,2}}\ldots p_{i,D}^{n_{i,D}}\;\;\;\;\;\;\;\forall \;i \end{array}$$

(2) $$\sum_{j=1}^{D}n_{i,j}\;=T\;\forall \;i$$

where $p_{i,j}$ is the probability of the pair j in loop i activated first or last, $n_{i,j}$, is the number of times that pair j in loop i is stimulated first or last. Furthermore, a robust Maximum Likelihood Estimation (MLE) is used to determine the probabilities of $p_{i,j}$.

### 2.2. Multinomial MLE

Multinomial MLE can be estimated for each pair of electrodes j in loop i by maximizing the multinomial Maximum Likelihood function, which can be described as the following:

(3) $$\min_{p_{i,j}}\overset{M}{\underset{l=1}{\sum }}\left(\log T!-\overset{D}{\underset{j=1}{\sum }}\log n_{i,j}^{l}!+\overset{D}{\underset{j=1}{\sum }}n_{i,j}^{l}\log p_{i,j}\right)\;\;\;\;\;\;\forall \;i$$

(4) $$\mathrm{Subject}\;\mathrm{to}.\;\sum_{j=1}^{D}p_{i,j}=1\;\forall \;i,j$$

(5) $$p_{i,j}\in \left[lb_{F_{i,j}},\;ub_{F_{i,j}}\right]\;\forall \;i,$$

where (4) ensures that the sum of all probabilities add up to one, and (5) constrains the most likely scenario within a confidence interval. The choices of $lb_{F_{i,j}}$, $\;ub_{F_{i,j}}$ are described in the following section. It is obvious to show that the MLE of multinomial distribution $p_{i,j}{}^{*}$ is equal to $\frac{n_{i,j}}{T}$, for j ∈{1,…,D}. However, due to the uncertainty in the LATs and the irregular activities of electrical waves in the presence of colliding wave fronts, the value of $n_{i,j}$ changes with respect to samples of LATs. Particularly, when there is more than one focal source, $n_{i,j}$ varies significantly. As a result, finding a robust estimator of the probability is important given the uncertain patterns of electrical wave propagation. In this study, the Monte Carlo concept is used, where random samples are generated to account for the uncertainty in both the LATs and the random electrical propagations. Specifically, M sets of LATs are generated and used to find the optimal estimator. 

Next, we applied the method introduced by [31] to construct a confidence interval for the probability of $p_{i,j}$. Define $\;S_{i}\left(w_{1},\ldots ,\;w_{D}\right)$ = $E\left[\left(Z_{i,1},\ldots ,Z_{i,D}\right)\right]\;$ for i= 1, 2, where $Z_{i,j}$ $=\left\{F_{i,j}\left(1\right),\ldots ,F_{i,j}\left(T\right)\right\}$ is a collection of binary vectors of $F_{i,j}$ with 1 representing that the sensor is activated first/last and 0 otherwise, $w_{j}$ = $\left(w_{j,1},\ldots ,w_{j,T}\right)$ is a weight vector to be optimized. A robust probability boundary for $p_{i,j}$ can be obtained through the following optimization [31]:

(6) $$\begin{array}{c} lb_{F_{i}}/ub_{F_{i}}∶=min\;/\;max\;S_{i}\left(w_{1},\ldots ,w_{D}\right)\forall \;i\\ \mathrm{Subject}\;\mathrm{to}.-2\sum_{j=1}^{D}\sum_{t=1}^{T}\log\left(Tw_{j,t}\right)\leq \chi_{1,1-\alpha }^{2}\forall \;j,t\\ \sum_{t=1}^{T}w_{j,t}=\;1\;\forall \;j\\ w_{j,t}\geq \;0\;\forall \;j,t \end{array}$$

where $w_{j,t}\;$ is an optimal weight vector estimated by the above optimization. By obtaining w, the robust minimum and maximum value of $E\left[Z_{i,j}\right]$ can be obtained, which provides the upper and lower bounds of $p_{i,j}$. Hereby, the outcome of the robust optimization provides, at the 1−α confidence level, probability boundaries for each pair of electrodes on whether it is the first or last pair to be stimulated.

### 2.3. Tracking Propagating Patterns

*Hypothesis Test to Determine the First/Last Activated Pair.* Suppose $P^{*}{}_{F,i}=$ ($p_{i,1}{}^{*},\ldots ,p_{i,\;D}{}^{*})$ is the probability estimates obtained from optimization (3), where $p_{i,j}{}^{*}$ represents the probability of sensor j activates first in loop i. It is assumed that all pairs of electrodes have an equal chance to be activated first, and the probability a pair activates first is 1/D. The test statistic is defined as the ratio between optimal probability, $p_{j}{}^{*}$, and uniform probability 1/D, which is assumed to follow a standard normal distribution. If an electrode pair is not activated first, the probability should be zero, so the null hypothesis is defined as: $H_{0}$:$p_{ij}{}^{*}\;$= 0, and the alternative hypothesis is $H_{a}$:$p_{ij}{}^{*}\neq$ 0. $p_{ij}{}^{*}$ is considered significant when the *p*-value is less than the significance level of α. The same procedure applies to the probability that a sensor activates last. The significant probabilities of sensors activating first or last can suggest most probable propagation paths, which can be used in the later section to identify focal sources. The number of significant probabilities can also suggest the number of electrical sources. For example, two significant probabilities in $P^{*}{}_{F,i}$ indicate that there are more likely two sources producing electrical impulses.

**Figure 2:**
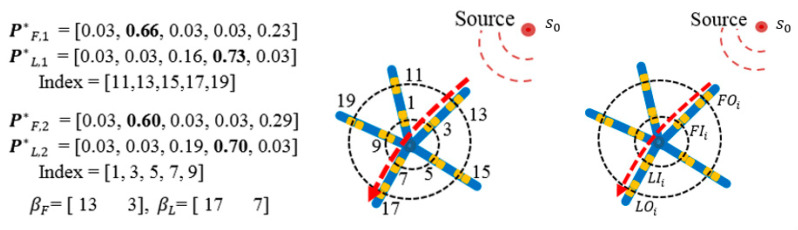
Identification of the most probable path.

*Most Probable Paths.* To explore the most probable paths that an electrical wave follows once it enters the outer loop (i=1) and then arrives at the inner circle (i=2), a vector $\beta_{F,\;i}$ is defined to record the index of the largest probability values in $P^{*}{}_{F,i}$. For example, as illustrated in Figure 2, the largest probabilities in $P^{*}{}_{F,1}$ and $P^{*}{}_{F,2}$ are 0.66 and 0.60, which correspond to the sensor index 13, and 3, respectively. The sensor indices are then recorded as $\beta_{F}$ = (13, 3). Similarly, given the last activated probability vector $P^{*}{}_{L,i}$, the most informative paths subject to being activated as the last sensor can be obtained following the same procedure as $\beta_{L}$ = (17, 7). Therefore, one of the most probable paths has been identified such that the electrical wave enters from node 13 and exits from sensor 17 (see the red dash line in Figure 2). In the case of more than one source, the same procedure can be implemented for the second largest values of probabilities belonging to $P^{*}{}_{F,i}$ and $P^{*}{}_{L,i}$ to identify the most probable paths from the second source.

*Focal Source Localization.* Assume that the most probable path for ith source is identified as *Path* = ($FO_{i},\;FI_{i},LI_{i},LO_{i})$, where $FO_{i}$, $\;FI_{i}$ are indices of first activated sensors in outer and inner loops, respectively. $LO_{i},\;LI_{i}$ are indices of last activated sensors in outer and inner loops, respectively (as seen in Figure 2 right). The activation time, $x_{FO_{i}}^{k}$, of $FO_{i}$ in kth activation generated by source i can be approximated using the following equation:

(7) $${\hat{x}}_{FO_{i}}^{k}=Geo_{i}\left(s_{0},\;s_{FO_{i}}\right)/CV^{*}+t_{i}^{k}+\varepsilon \;\;\;\;\;\forall \;j,k$$

where $Geo_{i}\left(s_{0},\;s_{FO_{i}}\right)$ is the geodesic distance between source $s_{0}$ and sensor $s_{FO_{i}}$, and $CV^{*}\;∿\;N\left(\mu ,\;\sigma_{c}^{2}\right)$ is the conduction velocity, which is assumed to follow a normal distribution with unknown parameters μ and $\sigma_{c}^{2}$. Note that $t_{i}^{k}$ is the activation time of a source with respect to $x_{FO_{i}}^{k}$. Similarly, the activation time of other sensors $s_{FI_{i}}$, $s_{LI_{i}}$, and $s_{LO_{i}}$ can be calculated in the same way. The coordinates of the source location $s_{0}$ can be obtained through an optimization to minimize the estimation error in (7). The process starts with collecting $T^{*}$ samples (i.e., activations), and the optimization is performed to minimize the sum of squared errors between the estimated and the true activation time for all four sensors of $s_{FO_{i}}$, $s_{FI_{i}}$, $s_{LI_{i}}$, and $s_{LO_{i}}$ as:

(8) $$\begin{array}{c} min\sum_{k=1}^{T^{*}}\sum_{e_{i}}{\left(\frac{Geo_{i}\left(s_{0}\;,\;\;\;s_{e_{i}}\right)}{CV^{*}}+t_{i}^{k}-x_{e_{i}}^{k}\right)}^{2}\\ \mathrm{Subject}\;\mathrm{to}.\;t_{i}^{k}<x_{FO_{i}}^{k}\;\forall \;k,i\\ D_{i}\leq \;\rho_{k}\;\forall \;k\\ D_{i},\;t_{i}^{k}\geq 0\;\forall \;k \end{array}$$

where $e_{i}$ represents the index of sensors in the most probable path, i.e., $FO_{i},\;FI_{i},LI_{i},LO_{i}$, $\rho_{i}$ is the maximum possible distance of the source to sensors, and $T^{*}$ represents the total number of samples matches the most probable path. The optimization repeats for *M* sets of samples, and different coordinates of sources locations can be estimated by Equation (8), where the average of estimated points provides a prediction with respect to focal source location. This method should be implemented for multiple locations of the catheter to ensure a robust estimation.

*Conduction Velocity Estimation.* One can assume the most probable path is reasonably close to the actual wave propagation with some errors and uncertainties. In this study, the true CV is estimated form a normal distribution with the parameters μ and $\sigma_{c}^{2}$. Since four pairs of sensors measure the travel path of an electrical wave, one can compute $\left(\begin{array}{c} 4\\ 2 \end{array}\right)$ = 6 different CVs with respect to each pair combination from the path based on their Geodesic distance and time differences. The mean μ and the standard deviation $\sigma_{c}$ of CV can be estimated as the average and standard deviation of the six estimated CVs from the four sensors. Finally, to insert the value of $CV^{*}$ into (8), one can randomly sample from $N\left(\mu ,\sigma_{c}^{2}\right)$ to calculate the estimated coordinates of the focal sources. 

## 3. Design of Experiments

The proposed method was applied and validated using simulated data at four scenarios where the focal sources were placed at four different locations of left atrium. The simulation model was developed using a 3D surface mesh of left atrium constructed from CT images of a normal heart [32]. The Courtemanche-Ramirez-Nattel (CRN) model was used to describe the electrical activities of human atrial myocytes [33], and the mono-domain tissue model was used to simulate electrical wave propagation. The PentaRay catheter was simulated, and the EGM at each electrode was calculated as the integral of ionic current normalized to the distance between the electrode and atrial myocytes [34]. Bipolar EGM was calculated as the difference of EGMs from each two pair of electrodes, which generates 10 leads of EGM signals. Each simulation ran for 4000 ms. The simulated EGMs, left atrium, and catheter are illustrated in Figure 1. 

Abnormal electrical impulses are generated irregularly at each source location to interrupt the normal sinus rhythm. For each case, the catheter is placed at 16 different locations. The focal source location is identified using the proposed method, and the identified area containing the source location was compared to the true location to evaluate algorithm performance. Experiments are performed using MATLAB 2018b on a 64-bit operating system.

## 4. Results

The proposed method was applied to detect focal sources using simulated EGM signals for four different scenarios. For each scenario, the catheter was placed at sixteen different locations to identify both normal and abnormal sources. As shown in Figure 1, triggers were placed close to the four pulmonary veins one at a time (see the red dots in Figure 1), and the normal source was set at the intersection between the atrial wall and the Bachmann’s bundle that conducts electrical impulses from the sinoatrial (SA) node to the left atrium (see the yellow dot in Figure 1). Figure 1 shows the examples of simulated EGM signals collected from three pair of electrodes (green dots in Figure 1). The simulation considered both the colliding of two wave fronts and spiral waves in the electrical propagation, and a video clip of electrical wave propagation is provided in the Video S1. In this section, we will first use one set of EGM data collected from one of the four scenarios to demonstrate the proposed method, which is followed by experimental results for all four scenarios.

### 4.1. MLE of Activation Probability

As discussed in Section 2, activations of electrodes follow a multinomial distribution, and the probability of each pair of electrodes activate first or last in both inner and outer loops can be calculated through the MLE problem defined in Equation (3). To implement the MLE, 30 activations were randomly selected from all the activations, and the number of events (i.e., a pair of electrodes is activated first/last) for each sensor pair was recorded as $n_{i,j}$, i = 1,2, j = 1, …, 5, where i was an index indicating the inner or the outer loo*p*, j was the index of the sensor pair. To eliminate the impacts of noise and uncertainty, sampling was done for 100 times (M = 100), and each sample contained 30 trials (T = 30). In addition, the hypothesis test was done to identify the first/last activated pairs in each loop.

**Figure 3:**
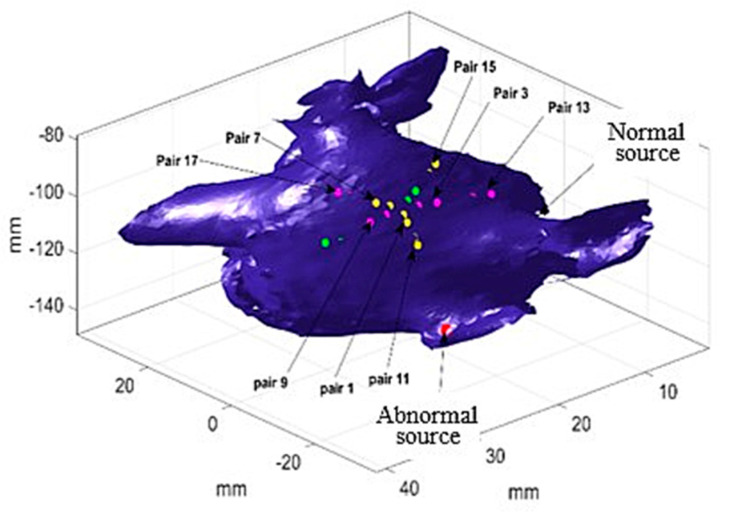
Two different electrical propagation paths in case study 1.

Table 1 shows the probabilities and *p*-values of each sensor pair activates first (column $P^{*}{}_{F,i}$) and last (column $P^{*}{}_{L,i}$) in outer loop and inner loop of the catheter. As seen in the second column of Table 1, there are two probabilities that are significantly large with small *p*-values, which suggests there are two sources generating electrical waves from two distinct locations. In this case, pair 11 and 13 (see the sensor index in Figure 3) in the outer loop with probabilities 0.31 and 0.42, respectively, are the first activated sensors. Pair 11 in the outer loop is activated first when one of the sources generates an electrical wave, which implies that pair 11 likely has shortest distance to the source. Likewise, pair 13 in the same loop is activated first when the other trigger sends an electrical signal. So, possibly, pair 13 in the outer loop is close to the second source. Similar results are obtained for the inner loop, where two electrode pairs, i.e., pair 1 and 3, have larger probabilities of 0.3 (*p*-value 0.07) and 0.45 (*p*-value 0.01), respectively. This shows the electrical waves that enter from pair 11 and 13 of the outer loop, respectively, arrive at the inner loop from pair 1 and 3, accordingly. A similar argument is held in the case of last activated pairs (See the third column of Table 1), where pairs 15 and 17 in the outer circle and pairs 7 and 9 of the inner circle are the termination nodes of the two different electrical waves in the two loops. This can be used to find indices related to the most probable paths, which, in this case, are 11-1-7-15 and 13-3-9-17, respectively. The most probable paths are used in the following section to identify the source locations.

### 4.2. Trigger Location Estimation

**Figure 4:**
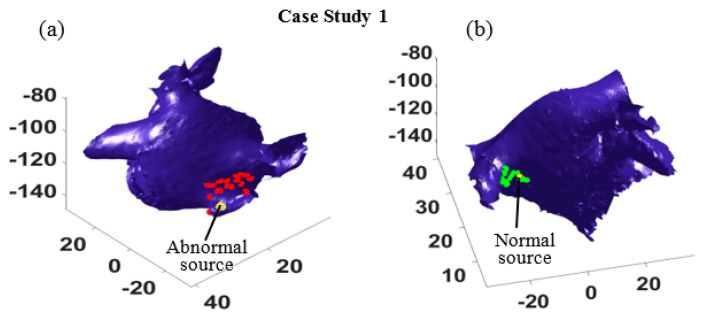
The estimated source locations from 16 different catheter locations in case study 1. (**a**) The red dots show the estimated locations of abnormal source, and the yellow dot shows the true location (**b**) The green dots mark the estimated locations of normal source, and the yellow dot marks the true location.

Given the most probable path in Table 1, one can estimate the locations of the two sources using the optimization defined in Equation (8). In our experiments, 30 activations (T^*^ = 30) were randomly selected to minimize the impact of uncertainties, and the source locations given EGM measured at sixteen different locations in the left atrium were identified (Figure 4). The red dots in Figure 4a show the estimated abnormal electrical source locations, and the green dots in Figure 4b show the estimated normal source locations. The estimations surround both sources and suggest regions of source locations, which demonstrates the accuracy of the proposed method.

### 4.3. Additional Experiments

**Figure 5:**
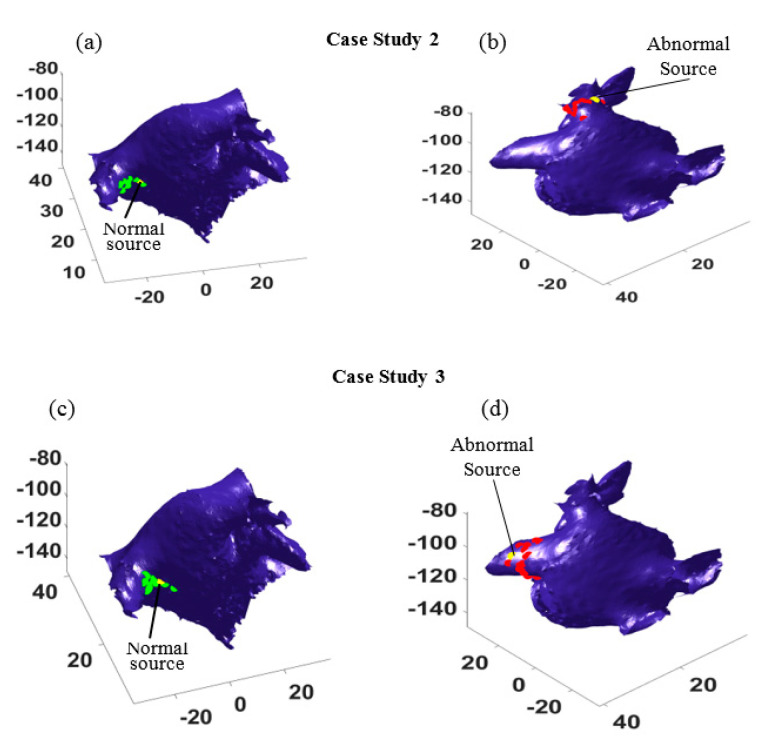
(**a**,**c**) show the estimated normal sources (the green dots) from 16 different catheter positions and their true locations (the yellow dots) in case study 2 and case study 3, respectively; (**b**,**d**) show the estimated abnormal sources (the red dots) from 16 different catheter positions and their true locations (the yellow dots) in case study 2 and case study 3, respectively.

To test the performance of the proposed method, more experiments were done for the other three different scenarios, where the focal source was placed close to the left superior (case study 2), left inferior (case study 3), and right superior (case study 4) pulmonary veins, respectively. In all the cases, there were sixteen independent experiments such that in each case the location of the catheter varied at sixteen different locations. This led to different probable paths that could be used to identify the source locations. The results for case studies 2 and 3 are shown in Figure 5, where the yellow dots show the locations of abnormal electrical source and normal source. The estimated source locations from sixteen different catheter locations are marked by green dots for the normal source and by red dots for the focal trigger. As seen in Figure 5, most of the estimations surround the true trigger locations. In both case studies, the estimated locations (see the green dots and red dots in Figure 5a,c and Figure 5b,d) distribute around the normal sources and the focal points (yellow dots). 

**Figure 6:**
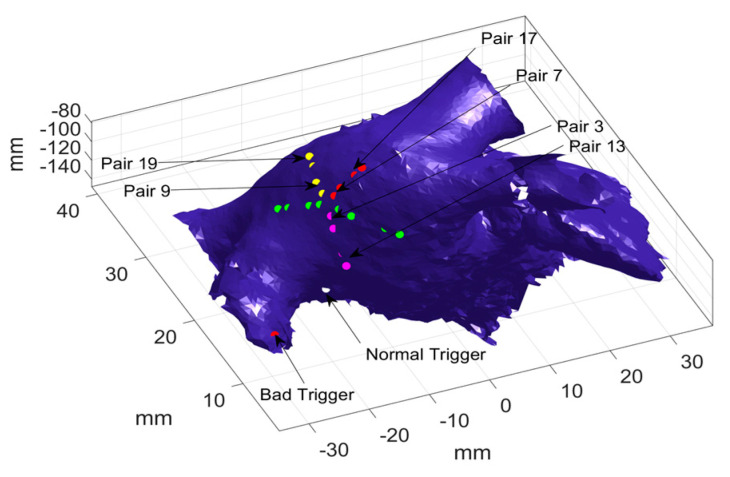
Electrical propagation paths when the abnormal source is near the normal source.

In addition, more experiments were performed to test the efficacy of proposed algorithm when the abnormal source was placed close to the normal source. As seen Figure 6, the abnormal trigger was given in the right superior pulmonary vein, which is near the normal source. This makes trigger localization more challenging as the two sources will generate the electrical impulses that propagate toward the same direction. We applied the proposed algorithm and the results are presented as follows.

Taking one catheter location as an example, optimization was performed to calculate the probability of each pair of electrodes being activated first or last, and the results are presented in Table 2. As shown in Table 2 column 2, pair 3 and 13 have significantly large probabilities (i.e., 0.89 and 0.87, respectively) as compared to the other pairs, which indicates the two pairs were activated first in most activations. It should be noted that only one significantly large probability was observed in each loop, which suggests that there is only one electrical source. Furthermore, Table 2 (see column 3) also shows that pair 7 and 17 have the largest probability (i.e., 0.58 and 0.74) of being activated last in the inner loop and outer loop, respectively. In addition, pair 9 and 19 have the second largest probability of being activated last, i.e., 0.37, and 0.2, respectively. This indicates two most probable paths, which means there are possibly two different electrical sources. Figure 6 illustrates the two most probable paths, i.e., 13-3-7-17, and 13-3-9-19, where the shared path (i.e., 13-3) is marked with pink dots and the distinguish path are marked with red (i.e., 7-17) and yellow (i.e., 9-19) dots, respectively. Given the most probable paths, the coordinates of the unknown trigger locations are calculated to identify the source locations.

**Figure 7:**
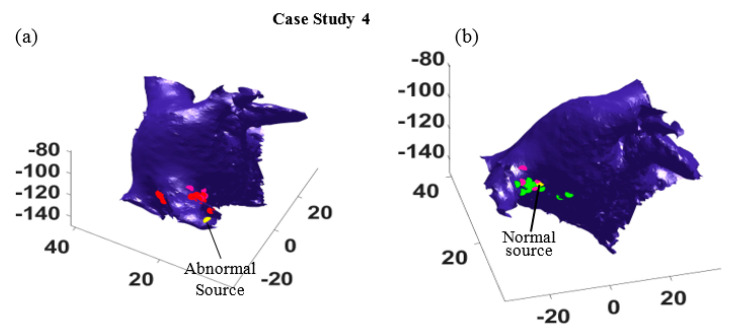
The estimated locations of abnormal (**a**) and normal (**b**) sources at 16 different catheter positions in case study 4. (**a**) The red dots mark the estimated abnormal sources and the yellow dot marks the true location. (**b**) The green dots mark the estimated normal sources and the yellow dot marks its true location.

Furthermore, the most probable paths for all sixteen different catheter locations were identified, and the location of the abnormal source and the normal source are estimated. In Figure 7, the actual locations of the abnormal and normal sources are shown by yellow dots, and the estimated abnormal and normal sources are marked by red dots and green dots, respectively. As seen in Figure 7, the estimations are all distributed around the right superior pulmonary vein, and some estimation correctly find the normal (Figure 7a) and the abnormal source (Figure 7b). It is worth mentioning that for some catheter locations and orientation, the algorithm cannot distinguish the travel path of electrical waves generated from the two sources, because these waves are propagating in the same directions. In such cases, only one path is identified, which provides one estimation of the source location (see the pink dots in Figure 7b).

## 5. Discussion

This study introduces a new method to account for different sources of uncertainties in abnormal electrical impulse identification due to the local activation time (LAT) estimation, the conduction velocity (CV) approximation, and the stochastic and chaotic activities of electrical waves in arrhythmias. 

*Uncertainty in LAT*. LAT is often obtained from the complex and noisy fractionated electrograms. It is difficult to identify an exact estimation of LAT, and the estimations can involve uncertainty. The proposed algorithm used a normal distribution to approximate the estimation error, and further propagated the error into the detection steps. Specifically, the true LAT is assumed to follow a normal distribution centered at an estimated LAT, and multiple samples of true LAT are generated and used in the detection algorithm. Normal distribution is chosen as it is commonly used to model estimation errors in the literature. However, LAT uncertainty can be quantified with different distributions upon validations using real data and computational experiments. The present study aims to introduce the analytical framework to account for LAT uncertainty in the electrical source identification, hence the choice of LAT distributions will not be discussed in this paper in the interests of brevity.

*Uncertainty in conduction velocity*. Using only a geometric approach for CV estimation can be prone to some uncertainty. In [24], the authors introduced a new technique to estimate CV, but the calculation is restricted to knowing the exact LAT, and assuming the wave front only follows a planar or circular wave. However, CV may vary between different waves in different activations and is sensitive to the measurement error regarding LAT. The proposed algorithm considers that the CV in a local region behind the catheter follows a distribution determined by the mean and standard deviation of the CVs estimated from multiple sensors. The true CVs are randomly sampled to consider the uncertainty associated with CV estimations. These CVs are further used to identify the sources of electrical waves. However, when electrical waves travel in cardiac tissue, their CV may vary due to fibrosis or muscle fiber heterogeneity, and the electrical wavefront could encounter different CVs. Using a local CV to approximate the global CV can greatly affect the detection accuracy. The proposed algorithm intends to reduce the impact of CV variations by placing sensors at multiple places and estimating the sources independently at each location. However, estimations can be sensitive to the distance between sensors and sources. When sensors are close to a focal source, the algorithm tends to provide a more accurate estimation. Therefore, future studies on optimal sensor placement can further improve the accuracy of the proposed algorithm.

*Uncertainty due to stochastic and chaotic activities of electrical waves in cardiac disorders*. The algorithm is tested with simulation data in the presence of spiral wave and wave breakups, which shows a robust detection performance. This is owing to the statistical nature of the proposed design. In this study, the algorithm does not rely on any single cycles of electrical conduction. Instead, it learns the dominate activity of electrical waves from their chaotic actions. For example, the multinomial MLE identifies the sensors that are most likely to be activated first or last among many activations. These activations may involve different propagation patterns leading to distinct travel paths. However, the optimization aims to find the maximum likelihood that a certain sensor is activated first or last. It should be noted that this study does not intend to identify all types of drivers, such as rotors and wave reentry. Rather, it aims to introduce a new statistical modeling concept for focal source identification, which is robust to spiral waves, wave breakup, noise, and other sources of uncertainty. 

*Catheter and triggers*. The proposed algorithm is validated through simulation study with two sources using a PentaRay catheter. However, it can be generalized to different types of catheters, such as a linear or HD Grid catheter. To adapt the algorithm to different catheters, one could define a multinomial distribution with more or fewer Bernoulli random variables to account for more or fewer sensors. In addition, the algorithm can determine the number of focal sources through the hypothesis test. Specifically, when the *p*-values of n sensors are significantly small, which indicates that the n sensors have higher chances of being activated first/last, it is identified that there are more likely n sources. 

Many methods have been introduced in the literature to analyze EGMs. These methods are generally in three categories: signal processing techniques such as dominant frequency analysis and recurrence quantification analysis [8,9,10,11,12,13,21,22], machine learning and pattern recognition techniques such as principle component analysis, independent component analysis, linear discriminant analysis and quadratic discriminant analysis [15,16,19,20], and geometric approaches that infer the travel paths of electrical waves using LATs [17,23,24,25,26,27]. The proposed method belongs to the geometric category, as it tracks the most probable travel path using LATs, and identifies the focal source based on geodesic distances. However, the proposed approach is different from existing methods in that it uses statistical modeling and robust optimization to infer the most probable path and is less sensitive to uncertainties in EGMs and LAT estimations. There are some similarities between the proposed method and the STAR method [35], both of which seek the dominant activations to identify driving sites. In STAR method, a portion of time in which a given electrode leads compared to the neighboring electrodes is used to rank the electrode. Then, this rank will be used to identify the AF site required for the ablation procedure. The STAR method identifies the regions that most frequently precede the activation of the neighboring area where one region is compared against the rest of the regions in each activation and the activation order of the rest of the regions is not evaluated. The proposed method estimates the probabilities of being activated first for all electrodes using a multinomial distribution and the probabilities for all electrodes are obtained through the robust optimization. In addition, STAR operates based on collected activation times from unipolar electrograms and does not consider estimation errors in LAT. The uncertainty associated with LAT estimation can propagate into the detection procedure. The proposed method accounts for multiple sources of uncertainty in EGMs to obtain a reliable estimation of electrical source. Lastly, STAR did not discuss how to deal with multiple drivers, while the proposed approach can determine the number of focal sources and distinguish multiple propagation paths. 

## 6. Conclusions and Limitations

This study introduces the concept of statistical modeling and robust optimization to account for different sources of uncertainty so as to realize a robust focal source localization. The designed algorithm uses a multinomial distribution to model the activation probability of each sensor and identifies the most probable travel path through a robust maximum likelihood estimation. The electrical source location is estimated from the most probable paths at different catheter locations. The method was tested and validated using simulation data at four different scenarios, where abnormal sources were placed at four distinct locations. The validation experiments simulated chaotic electrical wave propagation in the left atrium with one normal source and one focal source which caused wave collisions and spiral wave. The algorithm was applied at sixteen different catheter locations, which suggested the region that contains true focal sources locations. 

The estimation results are sensitive to the catheter configuration, i.e., for some sensor locations, the algorithm can precisely identify the true location, but, for some cases, the estimation is less accurate. The possible reasons can be that a local CV estimate is used for each activation. When the distance between the sensors and the focal source is large, i.e., the travel path of the electrical wave from the focal source to the catheter is long, the numerical error increases. This suggests a future study on dynamic sensor placement to navigate the catheter to maximize the benefit of the proposed algorithm. In addition, the proposed approach was tested using simulated EGMs, and future studies can be done to validate the method using clinical data. Despite the abovementioned limitation, the proposed algorithm exploits statistical modeling and optimization to quantify different sources of uncertainties involved in the electrical source localization procedure and provide a robust estimation of focal source locations.

## Figures and Tables

**Table 1:** Optimal probabilities for being activated first and last in case study one.

	$P^{*}{}_{F,i}$	$P^{*}{}_{L,i}$
Outer Loop	[0.31, 0.42, 0.09, 0.09, 0.05]	[0.07, 0.1, 0.27, 0.47, 0.06]
*p*-Values	[0.06, 0.02, 0.33, 0.33, 0.4]	[0.36, 0.31, 0.09, 0.01, 0.38]
Pair Index	11-13-15-17-19	11-13-15-17-19
Inner Loop	[0.3, 0.45, 0.07, 0.09, 0.06]	[0.08, 0.13, 0.03, 0.29, 0.45]
*p*-Values	[0.07, 0.01, 0.36, 0.33, 0.38]	[0.34, 0.26, 0.44, 0.07, 0.01]
Pair Index	1-3-5-7-9	1-3-5-7-9

**Table 2:** Optimal probabilities for being activated first and last in case study 4.

	$P^{*}{}_{F,i}$	$P^{*}{}_{L,i}$
Outer Loop	[0.02, 0.87, 0.07, 0.01, 0.01]	[0.01, 0.02, 0.01, 0.74, 0.2]
*p*-Values	[0.46, 0.01, 0.36, 0.48, 0.48]	[0.48, 0.46, 0.48, 0.01, 0.16]
Pair Index	11-13-15-17-19	11-13-15-17-19
Inner Loop	[0.01, 0.89, 0.06, 0.01, 0.01]	[0.01, 0.02, 0.01, 0.58, 0.37]
*p*-Values	[0.48, 0.01, 0.38, 0.48, 0.48]	[0.48, 0.46, 0.48, 0.01, 0.03]
Pair Index	1-3-5-7-9	1-3-5-7-9

## Supplementary Materials

The following are available online at https://www.mdpi.com/2306-5354/7/2/62/s1, Video S1: Simulated electrical wave propagation, spiral waves, wave breakups caused by abnormal electrical impulses.
